# BMP5 silencing inhibits chondrocyte senescence and apoptosis as well as osteoarthritis progression in mice

**DOI:** 10.18632/aging.202708

**Published:** 2021-03-19

**Authors:** Yan Shao, Chang Zhao, Jianying Pan, Chun Zeng, Hongbo Zhang, Liangliang Liu, Kai Fan, Xin Liu, Bingsheng Luo, Hang Fang, Xiaochun Bai, Haiyan Zhang, Daozhang Cai

**Affiliations:** 1 Department of Orthopedics, Orthopedic Hospital of Guangdong Province, Academy of Orthopedics Guangdong Province, The Third Affiliated Hospital of Southern Medical University, Guangzhou, China; 2 The Third School of Clinical Medicine, Southern Medical University, Guangzhou, China; 3 Guangdong Provincial Key Laboratory of Bone and Joint Degeneration Diseases, Guangzhou, China

**Keywords:** osteoarthritis, chondrocyte, BMP5, senescence, p38/ERK

## Abstract

In this study, we using the *in vivo* destabilization of the medial meniscus (DMM) mouse model to investigate the role of bone morphogenetic protein 5 (BMP5) in osteoarthritis (OA) progression mediated via chondrocyte senescence and apoptosis. BMP5 expression was significantly higher in knee articular cartilage tissues of OA patients and DMM model mice than the corresponding controls. The Osteoarthritis Research Society International scores based on histological staining of knee articular cartilage sections were lower in DMM mice where BMP5 was knocked down in chondrocytes than the corresponding controls 4 weeks after DMM surgery. DMM mice with BMP5-deficient chondrocytes showed reduced levels of matrix-degrading enzymes such as MMP13 and ADAMTS5 as well as reduced cartilage destruction. BMP5 knockdown also decreased chondrocyte apoptosis and senescence by suppressing the activation of p38 and ERK MAP kinases. These findings demonstrate that BMP5 silencing inhibits chondrocyte senescence and apoptosis as well as OA progression by downregulating activity in the p38/ERK signaling pathway.

## INTRODUCTION

Osteoarthritis (OA) is a progressive degenerative joint disease that involves cartilage destruction, synovitis, osteophyte formation, and subchondral bone remodeling [1, 2]. Progressive degeneration and loss of articular cartilage is a key feature of OA [3]. Chondrocytes maintain cartilage homeostasis by secreting extracellular matrix (ECM) components [2]. OA is associated with increased breakdown of the extracellular matrix by the matrix-degrading enzymes such as matrix metalloproteinase 3 (MMP3), MMP13, and ADAMTS5 [4–6]. Moreover, articular cartilage degradation is associated with higher levels of inflammatory cytokines and reactive oxygen species (ROS), which promote oxidative stress and induce aberrant chondrocyte proliferation, hypertrophy, autophagy, apoptosis, and senescence [7, 8].

Several studies have reported that chondrocyte senescence regulates OA progression [9–13]. Cellular senescence is a stress response resulting in an irreversible arrest of proliferation, and is commonly observed in degenerative and hyperplastic age-related pathologies. OA-related chondrocytes exhibit increased levels of senescence-associated beta-galactosidase (SA-β-Gal) activity, telomere shortening, and accumulation of p16^ink4a^ [14]. Intra-articular injection of senescent chondrocytes impairs the ability of the mesenchymal stem cells (MSCs) to regenerate cartilage [15]. Moreover, local clearance of senescent cells in the articular cartilage attenuates joint degeneration in the mouse OA model [10]. These data strongly suggest that chondrocyte senescence is a key checkpoint for OA progression and plays a critical role in the whole joint degeneration during aging. However, the mechanism of chondrocyte senescence during OA is not fully understood.

Chondrocyte apoptosis positively correlates with the severity of cartilage destruction and matrix depletion in human osteoarthritic cartilage tissues [16]. IL-1β induces *in vitro* apoptosis of human or mouse chondrocytes, thereby suggesting a link between inflammation and chondrocyte apoptosis [17]. However, the relationship between chondrocyte apoptosis and senescence during OA is controversial. Few studies suggest that chondrocyte apoptosis occurs in response to higher levels of stress, whereas, senescence is a consequence of lower levels of damage in the cartilage tissues of OA patients [18, 19]. Song et al demonstrated that doxorubicin promotes senescence at low doses and apoptosis at high doses in MCF7 breast cancer cells [20]. Chen et al demonstrated that high H_2_O_2_ doses induced apoptosis, whereas, low H_2_O_2_ doses induced senescence in F65 and IMR90 human diploid fibroblasts [21]. Munoz-Espin et al reported that the switch from senescence to apoptosis in the embryonic stages of the p21 knockdown mice resulted in detectable developmental abnormalities [22].

Bone morphogenetic proteins (BMPs) are members of the transforming growth factor beta (TGF-β) superfamily, and play an important role in bone and cartilage formation [23]. BMP-7 and BMP-9 are the most widely studied BMPs that have been identified so far [24]. The G allele variant of the *BMP5* gene is significantly associated with knee OA susceptibility in the Chinese Han population [25, 26]. BMP5 overexpression increases the expression of hypertrophy markers in mice, thereby suggesting an important role of BMP5 in OA [27]. Furthermore, BMP5 inhibits growth and induces apoptosis in human myeloma cells [28]. This suggests that BMP5 may regulate cell cycle progression and cellular senescence. However, the regulatory role of BMP5 in OA progression has not yet been established. Hence, we investigated the role of BMP5 in chondrocyte senescence and apoptosis, and its subsequent effects on OA progression using the destabilization of the medial meniscus (DMM) mouse model of OA as well as *in vitro* experiments using IL-1β-induced murine chondrocytes.

## RESULTS

### BMP5 is overexpressed in the chondrocytes from the knee articular cartilage of patients with OA and DMM-OA model mice

**Figure 1:**
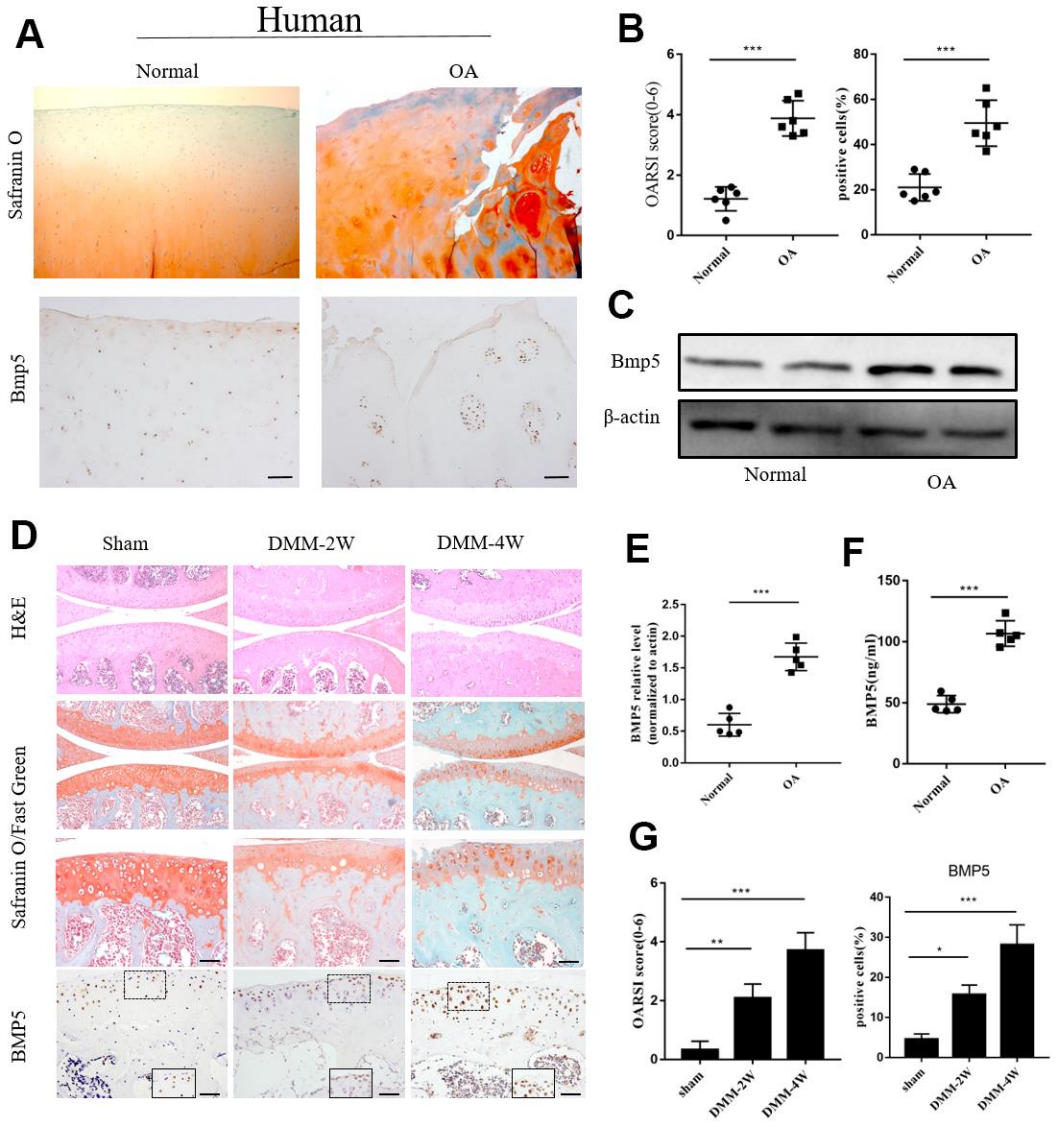
**Bmp5 is overexpressed in knee articular cartilage tissues of OA patients.** (**A**) Representative images show Safranin O-Fast Green staining and BMP5 immunohistochemical staining in the knee articular cartilage tissues from healthy subjects and OA patients (n=6 each). Scale bar: 10 μm. (**B**) OARSI scores based on the histochemical staining and corresponding BMP5 immunohistochemical staining of human knee articular cartilage tissue sections from healthy and osteoarthritis patients. (**C**) Representative western blots show BMP5 protein expression in the knee articular cartilage samples from healthy individuals and OA patients. (**D**) Representative images show Safranin O-Fast Green staining and BMP5 immunohistochemical staining in the knee articular cartilage sections from sham, DMM-2W and DMM-4W model mice (n=5 each). Scale bars: 10 μm (first line) and 5 μm (others). (**E**) Representative western blots show the levels of BMP5 protein in the knee articular cartilage sections from sham, DMM-2W and DMM-4W model mice. (**F**) ELISA assay results show joint fluid BMP5 levels in the healthy and OA patients. (**G**) BMP5 immunohistochemical staining scores and OARSI scores of the DMM-2W, DMM-4W, and sham model mice are shown. All data are represented as means ± SD.

We investigated the potential role of BMP5 in OA by analyzing knee articular cartilage tissues samples and joint fluid from OA patients that underwent total knee arthroplasty surgery and healthy patients that underwent amputation due to trauma. Immunohistochemistry (IHC) and western blotting analyses showed that BMP5 protein levels were significantly higher in the knee articular cartilage tissues from OA patients compared to those from the healthy subjects (Figure 1A–1C, 1E). ELISA results showed that the levels of BMP5 were higher in the synovial fluid of OA patients compared to those from healthy subjects (Figure 1F).

Next, we analyzed the levels of BMP5 in knee joint sections from DMM-2W (2 weeks after DMM surgery), DMM-4W (4 weeks after DMM surgery), and sham groups of mice. IHC analysis showed that BMP5 protein levels were higher in the knee cartilage sections from the DMM-4W and DMM-2W group mice compared to the sham-operated mice; moreover, BMP5 protein levels were higher in the DMM-4W mice compared to the DMM-2W mice (Figure 1D, 1G). Furthermore, BMP5 expression was significantly higher in the knee cartilage tissues from aged mice compared to those from young mice (Supplementary Figure 1A, 1B). These findings suggest that BMP5 overexpression may be involved in chondrocyte dysfunction and knee articular cartilage-related OA.

### BMP5 silencing delays OA progression in the DMM model mice

**Figure 2:**
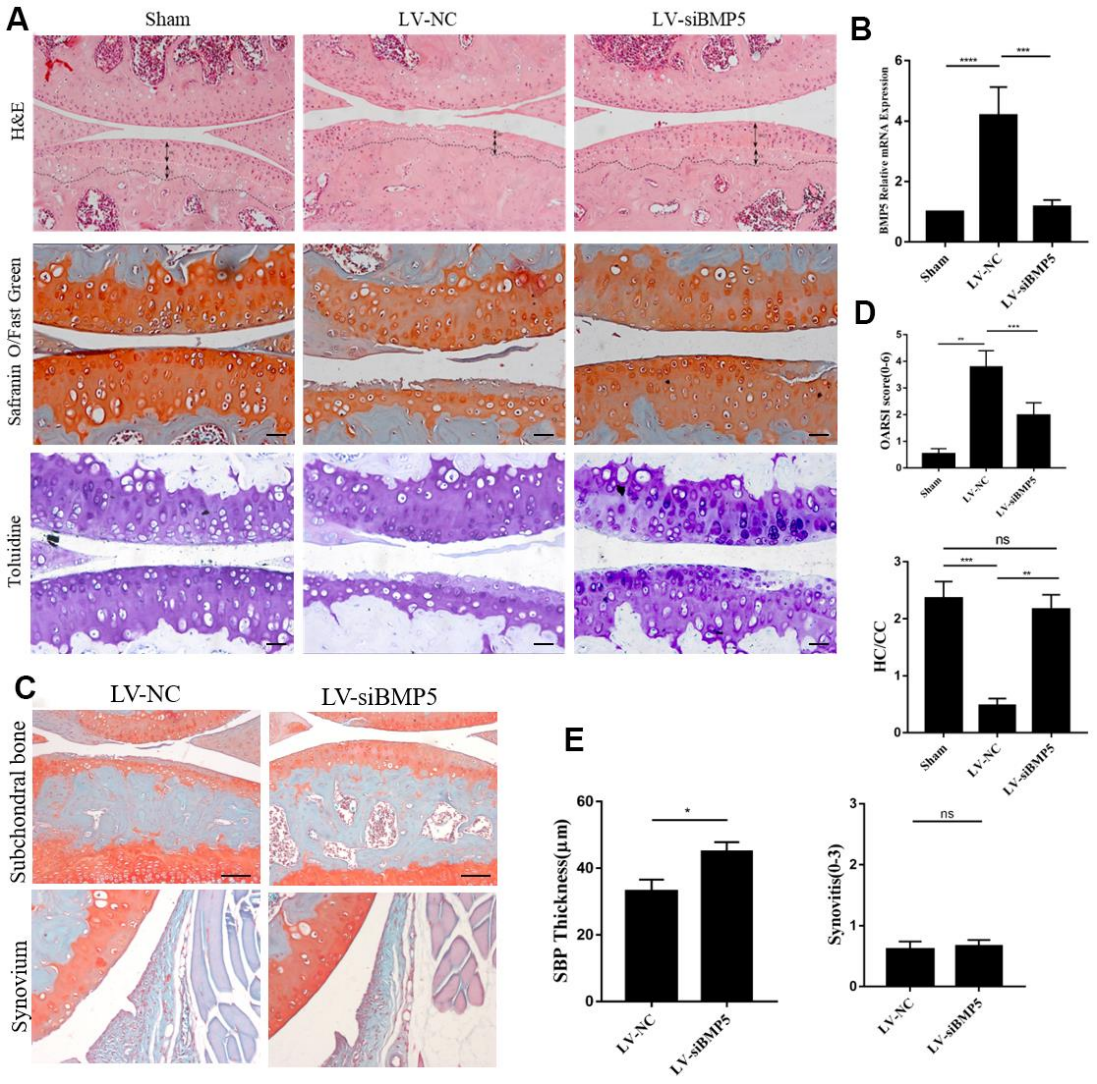
**Deletion of BMP5 attenuates osteoarthritis in the *in vivo* DMM model mice.** (**A**) Representative images show hematoxylin and eosin (H&E) staining, toluidine staining and Safranin O-Fast Green staining of knee cartilage tissue sections from LV-siBMP5 and LV-siNC group mice (n=5 per group). (**B**) RT-PCR results show BMP5 mRNA levels in LV-siBMP5 and LV-siNC group mice at 2 weeks post-DMM surgery (n = 5 per group). (**C**) Representative images show synovitis and SBP thickness in the sham-operated, DMM plus LV-siNC, and DMM plus LV-siBMP5 groups of mice at 4 weeks post-DMM operation. (**D**) OARSI scores and hyaline cartilage/calcified cartilage ratios in sham-operated, DMM plus LV-siNC, and DMM plus LV-siBMP5 groups of mice at 4 weeks post-DMM operation. (**E**) Quantitative analysis shows the status of synovitis and SBP thickness in the sham-operated, DMM plus LV-siNC, and DMM plus LV-siBMP5 groups of mice at 4 weeks post-DMM operation. All data are represented as the means ± SD (n=5 per group); scale bars: 5 μm.

To determine the potential role of BMP5 in OA development, we injected lentiviral-si-BMP5 (LV-si-BMP5) and LV-si-NC at 7 and 14 days after DMM surgery. We confirmed lentiviral transduction efficiency in the murine articular cartilage tissues by RT-PCR at 2 weeks after intra-articular injections (Figure 2B). BMP5 mRNA and protein levels were significantly reduced in the chondrocytes from the DMM + LV-siBMP5 group mice at four weeks after intra-articular lentiviral injections compared to those from the DMM + LV-si-NC group (Figure 2B and Supplementary Figure 2A, 2B).

Histological staining analysis of the knee cartilage specimens from DMM + LV-siBMP5 and DMM + LV-si-NC group mice is shown in Figure 2A, and the results of the OARSI scoring is shown in Figure 2D. At 4 weeks after DMM surgery, we observed reduced hyaline cartilage/calcified cartilage (HC/CC) ratio and the proportions of chondrocytes in the knee joint cartilage tissue and induced cartilage degradation, severe cartilage erosion, synovitis, and thickening of the subchondral bone plate (Figure 2A). However, compared to the DMM + LV-siNC group, the knee joint cartilage tissues from the DMM + LV-siBMP5 group mice showed reduced cartilage degradation (Figure 2A), lower OARSI grades (Figure 2D), and increased subchondral bone plate (SBP) at 2 and 4 weeks after DMM surgery (Figure 2C, 2E). Both LV-siNC and LV-siBMP5 groups showed similar levels of synovitis at 2 weeks and 4 weeks post-DMM surgery (Figure 2C, 2E). These results suggest that BMP5 promotes OA progression in the DMM model mice.

### BMP5 silencing reduces OA-related pathology in both *in vivo* and *in vitro* OA models by decreasing the expression of catabolic factors in the chondrocytes

Next, we investigated if BMP5 silencing affects the expression levels of matrix-degrading enzymes and aggrecanase in chondrocytes in order to determine the mechanisms through which BMP5 promotes cartilage destruction in OA. We transfected chondrocytes with multiple siRNAs against BMP5 (BMP5-siRNA1, BMP5-siRNA2, and BMP5-siRNA3) and observed that BMP5-siRNA1 significantly reduced BMP5 mRNA and protein levels in the chondrocytes compared to siRNA2-BMP5, siRNA3-BMP5 and si-NC (Supplementary Figure 3A, 3B). Hence we selected BMP5-siRNA1 for subsequent experiments.

**Figure 3:**
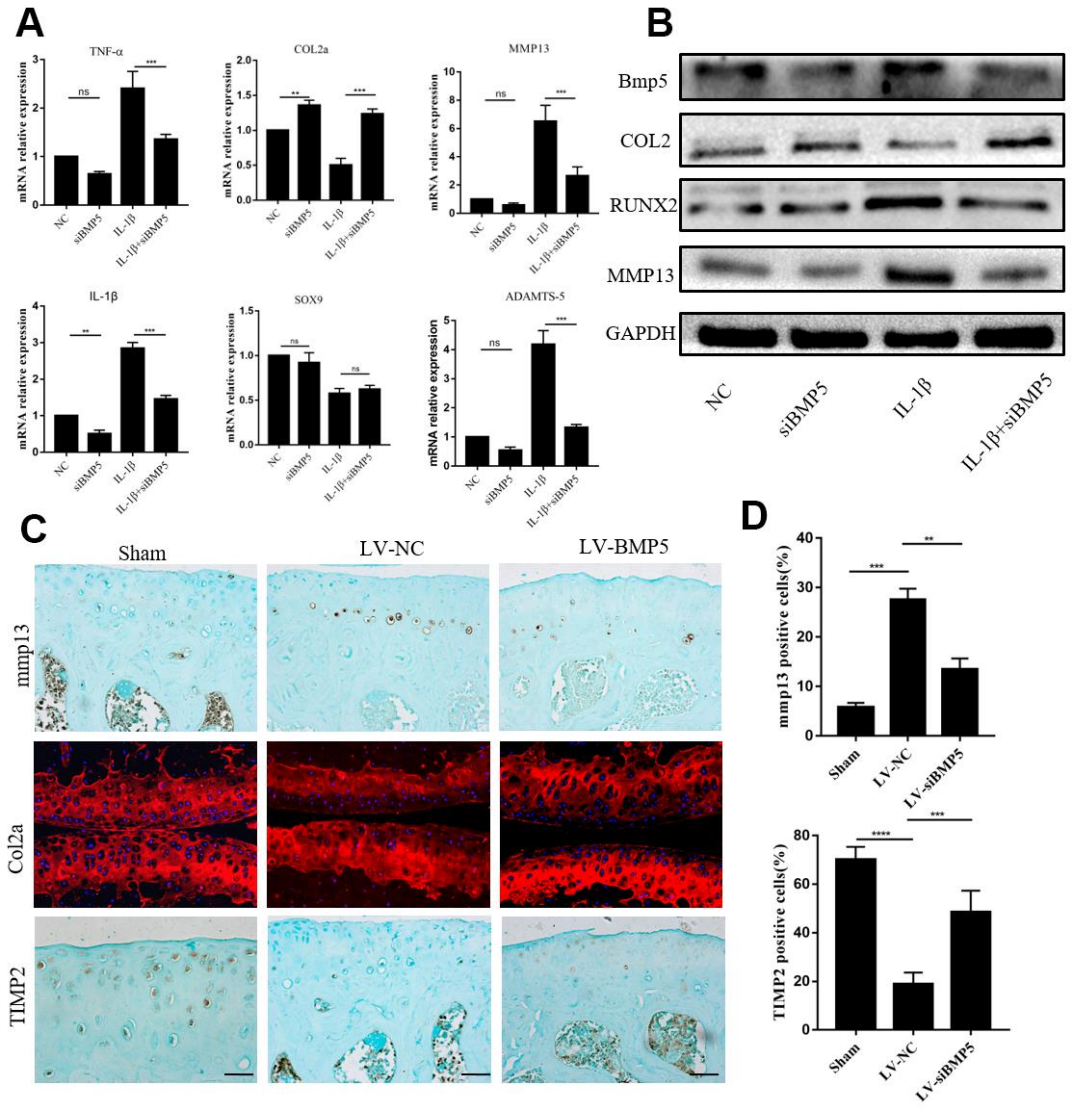
**BMP5 regulates *in vivo* and *in vitro* chondrocyte catabolism.** (**A**) QRT-PCR assay results show mRNA expression levels of pro-inflammatory cytokines (TNF-α, IL-1β), anabolic factors (type II collagen and SOX9), and catabolic factors (MMP13 and ADAMTS-5) in control and BMP-knockdown murine chondrocytes, treated with IL-1β or not. (**B**) Representative western blots show expression levels of MMP13, Runx2, and Col2 proteins in control and BMP-knockdown murine chondrocytes. (**C**) Representative immunohistochemical images and (**D**) quantitative analyses of the IHC results show MMP13, Col2a, and TIMP2 expression in the knee articular cartilage sections from sham-operated, DMM plus LV-siNC, and DMM plus LV-siBMP5 groups of mice at 4 weeks post-DMM operation. All data are represented as means ± SD (n=5 per group).

Previous studies in mice demonstrated that pro-inflammatory cytokines (TNF-α and IL-1β), anabolic factors (type II collagen and SOX9), and catabolic factors (MMP13 and ADAMTS-5) played critical roles in OA progression [29]. Therefore, we analyzed these factors in the chondrocytes from both *in vitro* and *in vitro* OA models. IL-1β-treated BMP-silenced chondrocytes showed significant reduction in the mRNA and protein levels of ADAMTS5 and MMP13, but there is no change in SOX9 mRNA compared to the IL-1β control group chondrocytes (Figure 3A, 3B). Moreover, we observed significant reduction in MMP13 levels and increased expression of Col2a and TIMP2 in the chondrocytes from the LV-siBMP5 group mice compared to those from the LV-si-NC group mice (Figure 3C, 3D). These results suggest that BMP5 silencing in the knee articular cartilage chondrocytes reduces pathological changes related to OA in the DMM-induced model mice by decreasing the expression of matrix catabolic factors.

### Silencing of BMP5 inhibits chondrocyte senescence in both *in vivo* and *in vitro* OA models

**Figure 4:**
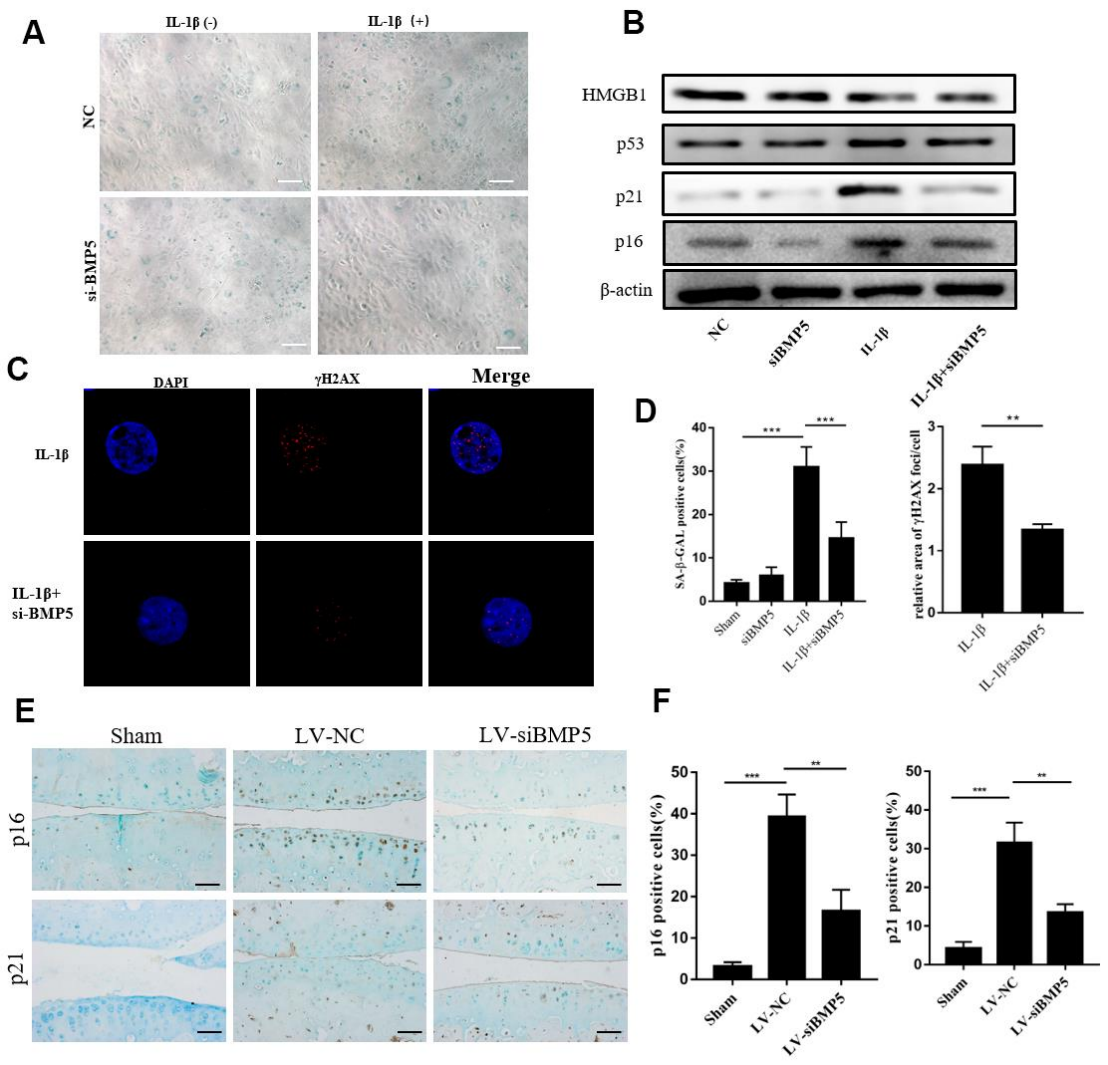
**BMP5 knockdown inhibits *in vitro* and *in vivo* chondrocyte senescence in the *in vitro* and *in vivo* OA models.** (**A**) The SA-β gal staining assay results in IL-1β-treated control and BMP5 knockdown murine chondrocytes. (**B**) Representative western blots show the levels of HMGB1, p53, p16, and p21 proteins in IL-1β-treated control and BMP5 knockdown murine chondrocytes. (**C**) Representative immunofluorescence images show γH2AX (red) staining in control and BMP5 knockdown murine chondrocytes treated with IL-1β. The chondrocytes were counterstained with DAPI (blue), a DNA binding dye. (**D**) Quantitative analyses show SA-β gal staining assay and γH2AX (red) staining in treated as above. (**E, F**) Representative immunohistochemical staining and quantitative analysis of p16 and p21 expression in the knee articular cartilage tissues from sham-operated, DMM plus LV-siNC, and DMM plus LV-siBMP5 groups of mice at 4 weeks post-DMM operation. All data are represented as the means ± SD (n=5); scale bars: 5 μm; **P<0.01; ***P<0.001.

We then performed SA-β-gal staining assay to determine the role of BMP5 in OA-related chondrocyte senescence. IL-1β-treated BMP5 knockdown chondrocytes showed significantly lower SA-β-gal activity than the corresponding controls (Figure 4A).

Furthermore, we assessed the protein levels of cyclin-dependent kinase inhibitors, p16, p21, p53, and HMGB1, which play a role in DNA damage-induced senescence [10, 21]. Western blotting analysis showed that p16, p21, HMGB1, and p53 protein levels were significantly reduced in the IL-1β-treated BMP5-silenced chondrocytes compared to the corresponding controls (Figure 4B).

Next, we analyzed γH2AX staining to determine if BMP5 knockdown reduced premature senescence of the chondrocytes. We observed that the relative area of γH2AX staining was significantly lower in the IL-1β-treated BMP5-depleted chondrocytes compared to the IL-1β-treated control chondrocytes (Figure 4C). We also demonstrated that p16 and p21 protein levels were significantly lower in the knee joint cartilage tissues from the DMM+LV-siBMP5 group mice compared to those from the DDM+LV-siNC group mice (Figure 4E, 4F). These data implied that BMP5 silencing reduced premature senescence of the chondrocytes in both *in vivo* and *in vitro* OA models.

### Depletion of BMP5 suppresses chondrocyte apoptosis in both *in vivo* and *in vitro* OA models

**Figure 5:**
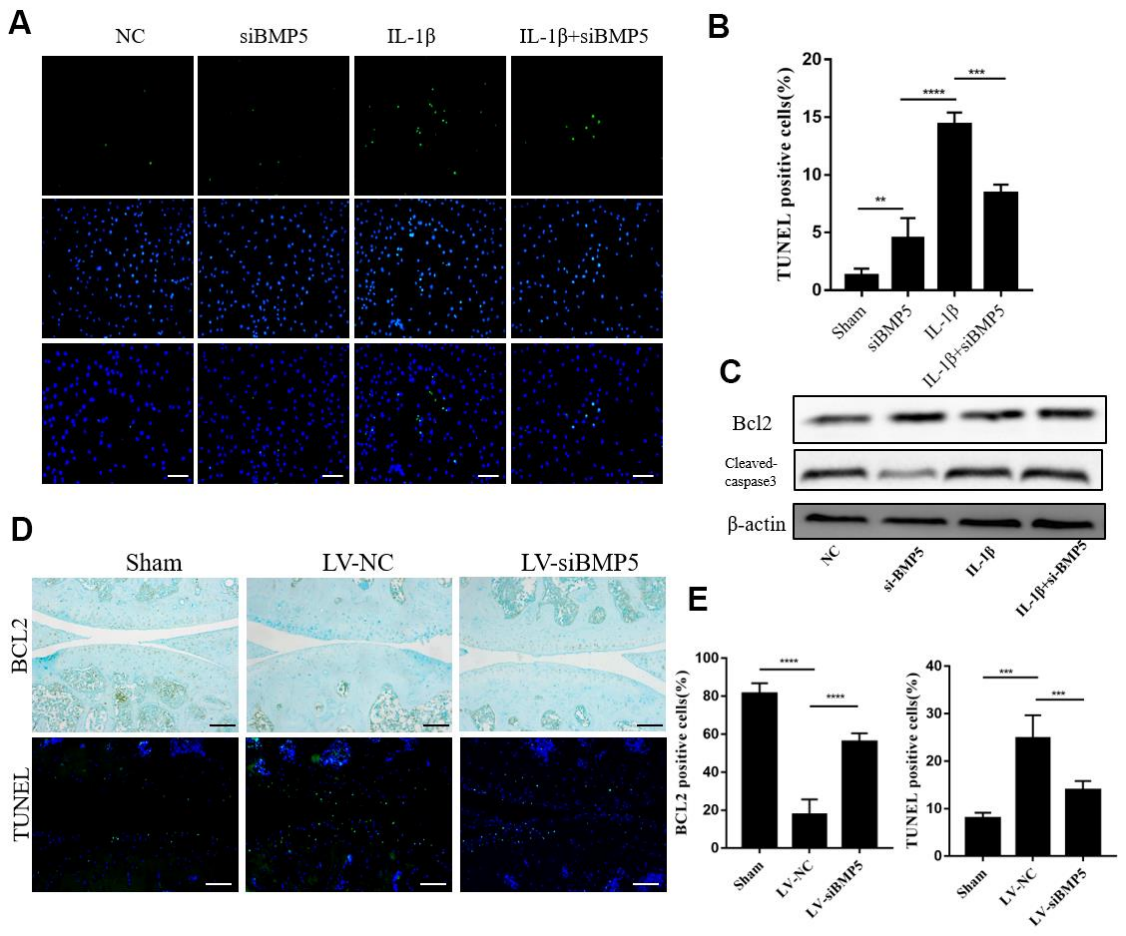
**BMP5 silencing protects against chondrocyte apoptosis in both *in vivo* and *in vitro* OA models. ** (**A**) Representative images show TUNEL staining assay in control and BMP5 knockdown murine chondrocytes treated with IL-1β. Scale bar: 5μm. (**B**) Quantitative analysis shows the total numbers of TUNEL-positive control and BMP5 knockdown chondrocytes treated with IL-1β. (**C**) Representative western blot images show BCL2 and cleaved-caspase3 protein levels in control and BMP5 knockdown chondrocytes treated with IL-1β. (**D**) Representative images and (**E**) quantitative analyses show TUNEL staining and BCl-2 immunohistochemical staining results in the knee articular cartilage tissues from sham-operated, DMM plus LV-siNC, and DMM plus LV-siBMP5 groups of mice at 4 weeks post-DMM operation. Scale bar: 5μm. All data are represented as the means ± SD (n=5); Scale bars: 5μm.

Chondrocyte apoptosis plays a significant role in articular cartilage destruction and matrix degradation in OA, an age-related degenerative human disease [16]. Therefore, we analyzed if BMP5 modulated the expression of anti-apoptotic or pro-apoptotic proteins in IL-1β-stimulated control or BMP5-silenced murine chondrocytes using western blotting, TUNEL staining, and flow cytometry assays. The numbers of TUNEL-positive cells were significantly reduced in the IL-1β-stimulated BMP5-silenced murine chondrocytes compared to the corresponding controls (Figure 5A, 5B). Furthermore, western blotting analysis showed that cleaved caspase-3 levels were significantly reduced and Bcl-2 levels were significantly increased in the IL-1β-stimulated BMP5-silenced murine chondrocytes compared to the corresponding controls (Figure 5C). Flow cytometry analysis confirmed that BMP-5 silencing reduced apoptosis in IL-1β-stimulated BMP5-silenced murine chondrocytes compared to the corresponding controls (Supplementary Figure 4A, 4B). The numbers of TUNEL-positive chondrocytes were significantly reduced in the LV-siBMP5 group compared to the LV-siNC group (Figure 5D, 5E). Conversely, BCl2 expression was significantly higher in the chondrocytes from the LV-siBMP5 group compared to those from the LV-siNC group (Figure 5D, 5E). These results demonstrate that BMP5 silencing reduced IL-1β-induced and DMM-induced chondrocyte apoptosis.

### BMP5 regulates chondrocyte senescence via p38/ERK signaling pathway

**Figure 6:**
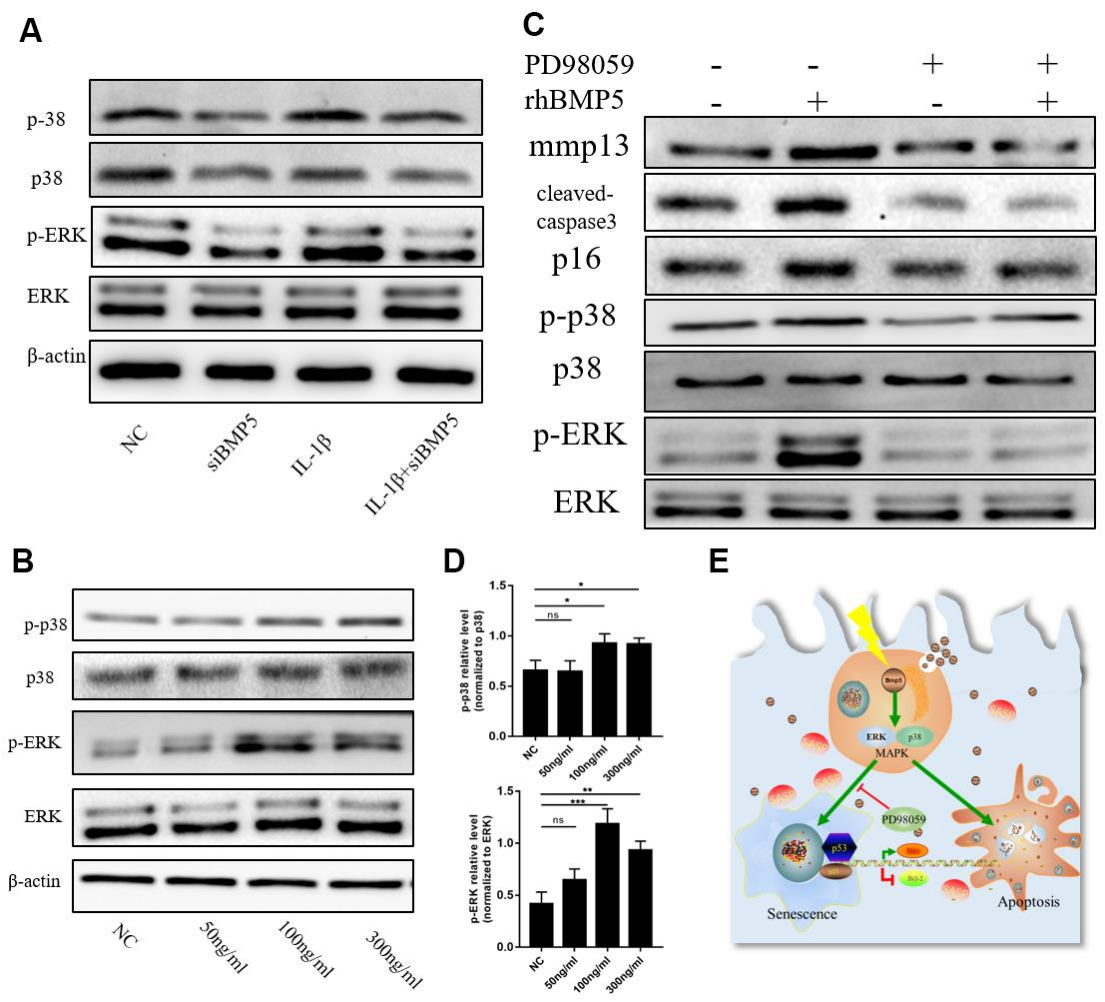
**BMP5 regulates osteoarthritis progression via p38/ERK signaling pathway.** (**A**) Representative western blots show the expression levels of total ERK, p-ERK, total p38, and p-38 proteins in control and BMP5 knockdown chondrocytes mouse chondrocytes treated with IL-1β. (**B**, **D**) Representative western blots and quantification show the expression levels of total ERK, p-ERK, total p38, and p-p38 in mouse chondrocytes only treated with different concentrations of rhBMP5(50,100,300ng/ml). (**C**) Western blot analysis shows the levels of total ERK, p-ERK, total p38, p-p38, p16, cleaved-caspase3, and MMP13 proteins in mouse chondrocytes treated with 100ng/ml rhBMP5, 10 μM PD98059 (MAPK inhibitor) or co-treatment with rhBMP5 and 10 μM PD98059. (**E**) A schematic working model shows the potential role of Bmp5 in regulating senescence and apoptosis of chondrocytes.

Next, we investigated the molecular mechanisms underlying BMP5 that regulate OA progression in the articular cartilage tissues. ERK1/2 signaling pathway plays a crucial role in promoting cellular senescence [30]. Therefore, we analyzed the status of the p38/ERK signaling pathway in control and BMP5-silenced chondrocytes subjected to IL-1β treatment by western blotting. The levels of total and phosphorylated p38 and ERK1/2 proteins were significantly higher in the IL-1β-treated control chondrocytes compared to the IL-1β-treated BMP5-silenced chondrocytes (Figure 6A). Moreover, the levels of p38 and ERK1/2 proteins were significantly increased in a dose-dependent manner by incubating chondrocytes with recombinant BMP5(Rocky Hill, NJ, USA, 120-39.), rhBMP5 (Figure 6B). Furthermore, IL-1β treatment of murine chondrocytes that were pre-incubated with 100ng/ml rhBMP5 and 10 μM PD98059 (inhibitor of ERK) showed reduced levels of total and phosphorylated p38 and ERK1/2 proteins, cleaved caspase-3, and p16 proteins compared to their corresponding controls (Figure 6C). These results demonstrate that BMP5 promotes chondrocyte senescence and apoptosis via p38/ERK signaling pathway.

## DISCUSSION

The role of BMP5 in OA is largely unknown. Snelling et al reported that BMP5 promotes chondrocyte hypertrophy [27]. Shih et al demonstrated that BMP5 was highly expressed in the neural crest progenitor cells and regulated cellular survival and proliferation via Smad and ERK signaling pathways, respectively; moreover, knockdown of BMP5 caused craniofacial malformation, but did not affect skeletal development [31]. Hence, in the present study, we investigated the role of BMP5 in OA progression using the DMM model mice.

The chondrocytes play a key role in cartilage metabolism. Functionally mature chondrocytes secrete collagen II (COL2) and glycosaminoglycans (GAGs) to maintain stable cartilage structure and function, and their dysfunction is associated with damaged cartilage during OA [3]. The cartilage-resident progenitor cells participate in cartilage repair by undergoing chondrogenic differentiation, whereas, the differentiated chondrocytes secrete the extracellular matrix (ECM) components and hence play a critical role in ECM anabolism [32]. Cartilage alterations in OA are mainly caused by imbalances in tissue remodeling because of changes in chondrocyte behavior [33]. During OA, ECM-degrading enzymes such as matrix metalloproteinases (MMPs) and a disintegrin and metalloproteinase with thrombospondin (ADAMTS) are overexpressed in the chondrocytes [4, 6]. In particular, collagen II (COL2) is a major target of MMP-13, whereas, ADAMTS is involved in aggrecan degradation [33]. Increased secretion of these ECM-degrading enzymes correlates with the matte appearance of the cartilage surface and exposure of the subchondral bone [34]. Moreover, inhibition of MMP-13 and ADAMTS attenuates progression of OA by decreasing the degradation of COL2 and aggrecan, thereby maintaining ECM homeostasis and structural integrity of the cartilage tissue [4, 6].

This study showed that BMP5 expression was significantly increased in the degenerative knee cartilage tissues of human OA patients and DMM-OA model mice. To further explore the role of BMP5 in OA, we knocked down BMP5 in chondrocytes using BMP5-siRNA transfections *in vitro* and intra-articular (IA) injections with lentiviruses carrying BMP5-siRNA *in vivo*. Histological staining results showed that the structure of the knee articular cartilage was more organized and stable in the LV-siBMP5 group mice compared to the LV-NC group mice. Immunohistochemical staining results showed that the levels of the main ECM degradation enzymes (ADAMTS5 and MMP13) in the knee articular cartilage tissue sections were significantly lower in the LV-siBMP5 group compared to the LV-NC group. Previous studies have shown that IL-1β promotes chondrocyte hypertrophy by activating the TGF/BMP signaling pathway, probably by inducing BMP5 expression [35, 36]. We observed that ADAMTS5 and MMP13 mRNA and protein expression levels were significantly reduced, but SOX9 mRNA and protein levels remained unchanged in IL-1β-treated BMP5-knockdown chondrocytes. Furthermore, *in vivo* BMP5 knockdown in the chondrocytes increased the levels of tissue inhibitor of metalloproteinase 2 (TIMP2) and decreased MMP13 expression.

We postulate that BMP5 knockdown probably decrease collagen degradation by inhibit MMPs, and increasing collagen II synthesis in the knee cartilage tissues of the LV-siBMP5 group mice compared to the LV-siNC group mice. Moreover, the activity of ADAMTS5, which regulates aggrecan degradation, is usually controlled post-translationally. We also observed that *in vivo* BMP5 silencing did not reduce ADAMTS5 mRNA levels in the chondrocytes. However, we observed significant differences in the aggrecan levels in the knee cartilage tissues from DMM plus LV-siNC and DMM plus LV-siBMP5 groups. Overall, these results showed that BMP5 silencing in the chondrocytes alleviates cartilage destruction by decreasing ECM degradation and increasing ECM synthesis.

The pathophysiology of OA involves multiple factors such as inflammation, mechanical stress, and metabolic alterations, all of which contribute to chondrocyte senescence [2, 7, 37]. Recent studies have reported that mesenchymal stem cells (MSCs) can be used to alleviate senescence and apoptosis of chondrocytes in osteoarthritis [38, 39]. We treated murine chondrocytes with IL-1β to stimulate hypertrophy and senescence *in vitro* and observed increased expression of γH2AX and p16INK4a as well as SA-β-gal levels, thereby demonstrating IL-1β-induced DNA damage, but these effects were abrogated by silencing BMP5. This suggests that BMP5 silencing protects chondrocytes from inflammation-induced senescence.

Chondrocyte senescence is associated with OA pathogenesis [13]. Previous studies showed that BMP5 overexpression inhibited cancer cell growth and arrested cells in the G1-phase of the cell cycle, thereby implying that BMP5 induced senescence by inhibiting the cell cycle [40, 41]. However, the role of BMP5 in chondrocyte senescence is not reported previously. In the present study, IL-1β-treated BMP5-silenced chondrocytes showed reduced SA-β-gal staining and lower levels of cellular aging-related proteins, p16 and p21. Moreover, the levels of the DNA damage and cellular senescence-related marker, γH2AX, is reduced in the BMP5-silenced chondrocytes, treated with IL-1β. These results suggest that BMP5 regulates subculture-induced chondrocyte senescence, which may be associated with OA pathogenesis.

Senescent chondrocytes show features of growth arrest as well as the senescence-associated secretory phenotype (SASP), which includes production of elevated levels of pro-inflammatory cytokines such as IL-6 and TNF-α as well as matrix-degrading enzymes such as MMP13 and MMP3, all of which play a significant role in OA [29, 42] Our study demonstrates that BMP silencing in the chondrocytes reduces the senescence-associated secretory phenotype (Figure 3A) inside the knee joint.

Previous studies have shown that chondrocytes undergo apoptosis because of increased levels of ROS and oxidative stress during OA [7, 43]. Excessive accumulation of ROS promotes release of activated mitochondrial apoptotic proteins and mitochondrial outer membrane permeabilization (MOMP), thereby activating cellular apoptosis [44]. In certain situations, mechanisms related to both senescence and apoptosis may co-operate to determine cell fate [45]. Repeated exposure to sub-cytotoxic levels of UVB rays [46] or treatment with low doses of H_2_O_2_ [21] induces senescence in human diploid fibroblasts. P53 is a critical senescent protein that activates intermediary caspase-2, which subsequently triggers executioner caspases-3, -6, and -9 [47]. We observed significantly reduced numbers of TUNEL-positive staining and cleaved caspase 3 levels in the BMP5-silenced chondrocytes treated with IL-1β. We also observed reduced TUNEL positive staining and increased BCL2 (anti-apoptotic protein) levels in the knee articular cartilage sections from the LV-siBMP5 group mice compared to those from the LV-NC group mice. These data suggest that BMP5 mediates chondrocyte senescence and apoptosis in the knee articular cartilage tissues during OA progression.

The p38/ERK1/2 signaling pathway regulates activation of chondrocyte-maturation genes and matrix degradation [48]. Therefore, we analyzed the relationship between BMP5 and the p38/ERK1/2 signaling pathway during OA. We observed that treatment of murine chondrocytes with rhBMP5 induced significantly higher levels of total and phosphorylated ERK1/2 and p38 protein levels. These effects were suppressed by pre-treatment with the MAPK inhibitor, PD98059. These results strongly suggest that BMP5 promotes OA via ERK/p38 signaling pathway.

In conclusion, our study shows that BMP5 is a novel and important regulator of chondrocyte senescence and OA progression via the ERK/p38 signaling pathway. Therefore, BMP5 is a potential treatment target for alleviating OA.

## MATERIALS AND METHODS

### Human knee cartilage samples

We obtained healthy human knee cartilage samples from patients undergoing amputation due to trauma (n=6) and OA cartilage specimens (n=6) from OA patients undergoing total knee joint arthroplasty at our hospital. The joint fluid was harvest from human osteoarthritic (n=5) and healthy (n = 5). This study was approved by the Ethics Committee of the third Affiliated Hospital of Southern Medical University. We also obtained informed consent from all patients before obtaining the cartilage samples.

### Cell lines, primary chondrocyte cultures, and siRNA interference

We isolated primary chondrocytes from the rib cartilage of newborn mice as previously described [49]. The primary chondrocytes were cultured in maintenance medium consisting of DMEM and nutrient mixture F12 (DMEM:F12; Gibco, Gaithersburg, MD, USA) supplemented with 20% fetal bovine serum (Gibco), 100 U/mL penicillin, and 100 mg/mL streptomycin (Gibco) in a humidified incubator maintained at 5% CO_2_ and 37° C. Primary chondrocytes were transfected with siRNA against BMP5 (BMP5-siRNA 1, 2 or 3) and control siRNA (si-NC) using Lipofectamine 3000 (Thermo Fisher Scientific, Waltham, MA, USA) according to the manufacturer's instructions. The medium was changed at 6 h after transfection. The BMP5 siRNA sequences were as follows:

siRNA1 forward primer: 5′- CCAGCCUACAUGAUACCAATT-3′;

siRNA1 reverse primer: 5′- UUGGUAUCAUGUAGGCUGGTT-3′;

siRNA2 forward primer: 5′-GCUGGGUUCAAGUGGGUUATT-3′;

siRNA2 reverse primer: 5′- UAACCCACUUGAACCCAGCTT-3′;

siRNA3 forward primer: 5′- CCAGGGAAACAAGCAUCUUTT-3′;

siRNA3 reverse primer: 5′- AAGAUGCUUGUUUCCCUGGTT-3′;

### Establishment of the DMM-OA model mice

We purchased thirty 12-week-old male C57BL/6J mice from the Experimental Animal Centre of Southern Medical University (Guangzhou, China). All mice were anesthetized by injecting chloral hydrate intraperitoneally followed by surgical destabilization of the medial meniscus (DMM) on the right knees to induce knee joint instability and posttraumatic osteoarthritis (PTOA) as previously described [50]. Sham surgery was performed by cutting the skin without exposing the knee joint capsule on the right knees of the control mice. DMM surgery was confirmed by using a stereo microscope to determine complete transection of the medial meniscotibial ligament (MMTL) and destabilization of the medial meniscus. The mice were randomly separated into three groups. The group 1 (n=5) animals included those that underwent sham operation and received intra-articular injection of phosphate-buffered saline; group 2 (n=5) animals included those that underwent DMM surgery and received intra-articular injection (6 μL) of 10^8^ TU/mL lentivirus-siNC (5’-3’ TTCTCCGAACGTGTCACGT); group 3 (n=5) animals included those that underwent DMM surgery and received intra-articular injection (6 μL) of 10^8^ TU/mL of lentivirus-siBMP5 (5’-3’ CCAGCCTACATGATACCAA). The lentiviruses carrying si-NC and si-BMP5 were obtained from GenePharma (Shanghai, China).

### Histological analysis

The mice were sacrificed at 2 or 4 weeks after DMM surgery. Knee joints were harvested, decalcified with 0.5 M EDTA (pH 7.4) for 4 weeks, and embedded in paraffin. Then, 4 μm thick knee joint tissue sections were cut in a sagittal orientation and subjected to hematoxylin and eosin (H&E), Toluidine Blue Staining Kit (Leagene, Beijing, China) and Safranin O-Fast Green staining. Briefly, sections were deparaffinized, rehydrated, and incubated with Weigert’s iron hematoxylin solution for 3 min. Then, the stained slides were rinsed in distilled water and incubated with 1% acid–70%alcohol for 15 s. The slides were then incubated with 0.02% aqueous Fast Green solution for 5 min followed by 1% acetic acid for 30 s. Then, the slides were rinsed with distilled water and incubated with 1% Safranin-O solution for 10 min. Subsequently, the slides were dehydrated, cleared, and mounted. The severity of cartilage degeneration was assessed using the OARSI osteoarthritis cartilage histopathology assessment system [51] as shown in Table 1.

**Table 1:** The recommended semi-quantitative scoring system.

**Grade**	**Osteoarthritis damage**
0	normal structure
0.5	Only loss of Safranin-O staining
1	small fibrillation without loss of normal structure
2	Vertical down to the layer below the superficial layer and some loss of surface cell
3	Vertical erosion to the calcified cartilage extending to <25% of the articular surface
4	Vertical erosion to the calcified cartilage extending to 25%-50% of the articular surface
5	Vertical erosion to the calcified cartilage extending to 50%-75% of the articular surface
6	Vertical erosion to the calcified cartilage extending to >75% of the articular surface

### Immunohistochemistry

The de-paraffinized knee joint sections from DMM and sham mice were incubated overnight at 4° C with primary antibodies against BMP5 (Santa Cruz Biotechnology, Santa Cruz, CA, USA), Runx2, Col2a, MMP13, Adamts5 (Abcam, Cambridge, UK), as well as p16 and p21 (Proteintech, Rosemont, IL, USA). The slides were then incubated with the secondary antibody (Jackson ImmunoResearch Laboratories, Baltimore Pike, USA, 1:200)) in blocking solution for 1 h at room temperature, and developed with 3,3′-diamino-benzidine (DAB, ZSGB-Bio, Beijing, China).

### Immunofluorescence staining

The knee joint sections from DMM and sham mice were incubated overnight at 4° C with primary antibodies against γH2AX (Cell Signaling Technology, Danvers, MA, 1:100) and Col2a1(Abcam, Cambridge, UK). Then, the sections were incubated with species-matched Alexa-488 or Alexa-594-labeled secondary antibodies (Life Technologies, Grand Island, NY, USA). Subsequently, the sections were mounted with PBS containing DAPI (Thermo Fisher Scientific), and photographed using a FluoView FV1000 confocal microscope (Olympus, Tokyo, Japan). The proportions of positively stained cells relative to the total number of cells were determined for each sample.

### Quantitative real-time PCR analysis

Total RNA was isolated from 90% confluent cells grown in 12-well plates using the TRIzol reagent (Life Technologies, Grand Island, NY. Equal amounts of RNA samples were reverse transcribed to generate cDNA using the Reverse transcription kit (Vazyme Biotech, Nanjing, China). The quantitative PCR (qPCR) assays were performed to determine the levels of Bmp5, TNF-α, IL-1β, SOX9, COL2A, MMP13, and ADAMTS mRNAs relative to GAPDH mRNA (internal loading control) using the Real-Time PCR Mix (Vazyme Biotech) in a Light Cycler (Roche Molecular Biochemicals, Indianapolis, IN, USA). The following primers were used for q-PCR:

BMP5 forward primer: 5′- CCACGTACCAAAACCTTGCT-3′;

BMP5 reverse primer: 5′- CAATGCTGTTAAGGCGAACA-3′;

TNF-α forward primer: 5′-ATGAGCACAGAAAGCATGA-3′;

TNF-α reverse primer: 5′-AGTAGACAGAAGAGCGTGGT-3′;

IL-1β forward primer: 5′- GCACTACAGGCTCCGAGATGAAC-3′;

IL-1β reverse primer: 5′- TTGTCGTTGCTTGGTTCTCCTTGT-3′;

SOX9 forward primer: 5′-AGTACCCGCATCTGCACAAC-3′;

SOX9 reverse primer: 5′-ACGAAGGGTCTCTTCTCGCT-3′;

ACAN forward primer: 5′- GGCAACCTCCTGGGTGTAAG-3′;

ACAN reverse primer: 5′- TGGGGTTCGTGGGCTCACAA-3′;

Col2a1 forward primer: 5′-CACACTGGTAAGTGGGGCAAGACCG-3′;

Col2a1 reverse primer: 5′-GGATTGTGTTGTTTCAGGGTTCGGG-3′;

MMP-13 forward primer: 5′-GCTGCGGTTCACTTTGAGAA-3′;

MMP-13 reverse primer, 5′-GGCGGGGATAATCTTTGTCCA-3′;

GAPDH forward primer: 5′- AAATGGTGAAGGTCGGTGTGAAC-3′;

GAPDH reverse primer, 5′- CAACAATCTCCACTTTGCCACTG-3′.

### Western blotting

Total protein was extracted from chondrocytes by incubating them for 5 min at 95° C in lysis buffer [62.5 mM Tris-HCl (pH 6.8), 10% glycerol, 2% SDS, 50 Mm DTT, and 0.01% bromophenol blue]. Equal amounts of protein lysates were separated by SDS-PAGE and transferred onto a nitrocellulose (NC) membrane (Bio-Rad, Hercules, CA, USA). The blots were incubated overnight at 4° C with primary antibodies against BMP5 (ABclonal, Woburn, MA, USA), RUNX2 (ABclonal), MMP13 (Proteintech), p16 (Proteintech), p53 (Proteintech), p21 (Proteintech), ERK (Cell Signaling Technology, Danvers, MA, USA), p-ERK (Cell Signaling Technology), p38 (Cell Signaling Technology), and p-p38 (Cell Signaling Technology). Then, the blots were incubated at room temperature for 30 min with the corresponding HRP-labeled secondary antibodies ((Jackson ImmunoResearch Laboratories, Baltimore Pike, USA). The blots were then developed with the Enhanced chemiluminescence kit (Millipore, Burlington, MA, USA) and visualized using the Image J software.

### Senescence-associated β-galactosidase (SA-β-Gal) assay

The chondrocytes were stained with the SA-β-Gal staining kit (Biovision, Milpitas, CA, USA) according to the manufacturer's protocol. SA-β-Gal-positive cells were counted in four randomly selected fields per treatment (n=5) in each sample.

### TUNEL staining

DNA damage was detected by TUNEL staining in primary chondrocytes or knee cartilage sections using the *In situ* cell death detection kit (Roche, Basel, Switzerland) according to the manufacturer’s instructions. The fixed cells or sections were stained for 30 min at 37° C and the nuclei were stained with DAPI. The slides were observed under an Olympus fluorescence microscope (Olympus). We randomly selected twenty-five fields in each slide and counted the numbers of TUNEL-positive cells relative to total number of cells in a field.

### Apoptotic flow cytometry assay

Chondrocytes were seeded into 6-well plates at a density of 1 × 10^5^ cells per well. Flow cytometry was performed to detect the apoptosis rates of chondrocytes using the Annexin V/PI apoptosis detection kit (BD Biosciences, Franklin Lakes, NJ, USA). Chondrocytes were washed twice cold PBS, and then resuspended in binding buffer and incubated with 5μl FITC-Annexin V and 5μl PI at room temperature for 15 min in the dark. Next, cell suspensions were determined by using the FACScan flow cytometry system (Becton Dickinson, San Diego, CA, USA).

### Statistical analysis

Statistical analysis was performed using the SPSS statistical software for Windows, version 20.0 (SPSS, Chicago, IL, USA). All data were expressed as means ± SD. All experiments were performed in triplicate. The differences between groups were analyzed using one-way analysis of variance (ANOVA). P<0.05 was considered statistically significant.

## Supplementary Material

Supplementary Figures

## References

[1] Hunter DJ, Schofield D, Callander E. The individual and socioeconomic impact of osteoarthritis. Nat Rev Rheumatol. 2014; 10:437–41. 10.1038/nrrheum.2014.44 24662640

[2] Hunter DJ, Bierma-Zeinstra S. Osteoarthritis. Lancet. 2019; 393:1745–59. 10.1016/S0140-6736(19)30417-9 31034380

[3] Roseti L, Desando G, Cavallo C, Petretta M, Grigolo B. Articular Cartilage Regeneration in Osteoarthritis. Cells. 2019; 8:1305. 10.3390/cells8111305 31652798 PMC6912428

[4] Little CB, Barai A, Burkhardt D, Smith SM, Fosang AJ, Werb Z, Shah M, Thompson EW. Matrix metalloproteinase 13-deficient mice are resistant to osteoarthritic cartilage erosion but not chondrocyte hypertrophy or osteophyte development. Arthritis Rheum. 2009; 60:3723–33. 10.1002/art.25002 19950295 PMC2832925

[5] Wang J, Wang Y, Zhang H, Gao W, Lu M, Liu W, Li Y, Yin Z. Forkhead box C1 promotes the pathology of osteoarthritis by upregulating β-catenin in synovial fibroblasts. FEBS J. 2020; 287:3065–87. 10.1111/febs.15178 31837247

[6] Glasson SS, Askew R, Sheppard B, Carito B, Blanchet T, Ma HL, Flannery CR, Peluso D, Kanki K, Yang Z, Majumdar MK, Morris EA. Deletion of active ADAMTS5 prevents cartilage degradation in a murine model of osteoarthritis. Nature. 2005; 434:644–48. 10.1038/nature03369 15800624

[7] Brandl A, Hartmann A, Bechmann V, Graf B, Nerlich M, Angele P. Oxidative stress induces senescence in chondrocytes. J Orthop Res. 2011; 29:1114–20. 10.1002/jor.21348 21284033

[8] Henrotin YE, Bruckner P, Pujol JP. The role of reactive oxygen species in homeostasis and degradation of cartilage. Osteoarthritis Cartilage. 2003; 11:747–55. 10.1016/s1063-4584(03)00150-x 13129694

[9] Vinatier C, Domínguez E, Guicheux J, Caramés B. Role of the inflammation-autophagy-senescence integrative network in osteoarthritis. Front Physiol. 2018; 9:706. 10.3389/fphys.2018.00706 29988615 PMC6026810

[10] Jeon OH, Kim C, Laberge RM, Demaria M, Rathod S, Vasserot AP, Chung JW, Kim DH, Poon Y, David N, Baker DJ, van Deursen JM, Campisi J, Elisseeff JH. Local clearance of senescent cells attenuates the development of post-traumatic osteoarthritis and creates a pro-regenerative environment. Nat Med. 2017; 23:775–81. 10.1038/nm.4324 28436958 PMC5785239

[11] Diekman BO, Sessions GA, Collins JA, Knecht AK, Strum SL, Mitin NK, Carlson CS, Loeser RF, Sharpless NE. Expression of p16^INK^ ^4a^ is a biomarker of chondrocyte aging but does not cause osteoarthritis. Aging Cell. 2018; 17:e12771. 10.1111/acel.12771 29744983 PMC6052464

[12] Rahmati M, Nalesso G, Mobasheri A, Mozafari M. Aging and osteoarthritis: Central role of the extracellular matrix. Ageing Res Rev. 2017; 40:20–30. 10.1016/j.arr.2017.07.004 28774716

[13] Loeser RF, Collins JA, Diekman BO. Ageing and the pathogenesis of osteoarthritis. Nat Rev Rheumatol. 2016; 12:412–20. 10.1038/nrrheum.2016.65 27192932 PMC4938009

[14] McCulloch K, Litherland GJ, Rai TS. Cellular senescence in osteoarthritis pathology. Aging Cell. 2017; 16:210–18. 10.1111/acel.12562 28124466 PMC5334539

[15] Cao X, Luo P, Huang J, Liang C, He J, Wang Z, Shan D, Peng C, Wu S. Intraarticular senescent chondrocytes impair the cartilage regeneration capacity of mesenchymal stem cells. Stem Cell Res Ther. 2019; 10:86. 10.1186/s13287-019-1193-1 30867061 PMC6416972

[16] Aigner T, Kim HA. Apoptosis and cellular vitality: issues in osteoarthritic cartilage degeneration. Arthritis Rheum. 2002; 46:1986–96. 10.1002/art.10554 12209500

[17] Zhao CQ, Liu D, Li H, Jiang LS, Dai LY. Interleukin-1beta enhances the effect of serum deprivation on rat annular cell apoptosis. Apoptosis. 2007; 12:2155–61. 10.1007/s10495-007-0137-x 17912642

[18] Musumeci G, Castrogiovanni P, Trovato FM, Imbesi R, Giunta S, Szychlinska MA, Loreto C, Castorina S, Mobasheri A. Physical activity ameliorates cartilage degeneration in a rat model of aging: a study on lubricin expression. Scand J Med Sci Sports. 2015; 25:e222–30. 10.1111/sms.12290 25039883

[19] Musumeci G, Loreto C, Carnazza ML, Martinez G. Characterization of apoptosis in articular cartilage derived from the knee joints of patients with osteoarthritis. Knee Surg Sports Traumatol Arthrosc. 2011; 19:307–13. 10.1007/s00167-010-1215-0 20644910

[20] Song YS, Lee BY, Hwang ES. Dinstinct ROS and biochemical profiles in cells undergoing DNA damage-induced senescence and apoptosis. Mech Ageing Dev. 2005; 126:580–90. 10.1016/j.mad.2004.11.008 15811427

[21] Chen QM, Liu J, Merrett JB. Apoptosis or senescence-like growth arrest: influence of cell-cycle position, p53, p21 and bax in H2O2 response of normal human fibroblasts. Biochem J. 2000; 347:543–51. 10.1042/0264-6021:3470543 10749685 PMC1220988

[22] Muñoz-Espín D, Cañamero M, Maraver A, Gómez-López G, Contreras J, Murillo-Cuesta S, Rodríguez-Baeza A, Varela-Nieto I, Ruberte J, Collado M, Serrano M. Programmed cell senescence during mammalian embryonic development. Cell. 2013; 155:1104–18. 10.1016/j.cell.2013.10.019 24238962

[23] Lin S, Svoboda KK, Feng JQ, Jiang X. The biological function of type I receptors of bone morphogenetic protein in bone. Bone Res. 2016; 4:16005. 10.1038/boneres.2016.5 27088043 PMC4820739

[24] Kang Q, Sun MH, Cheng H, Peng Y, Montag AG, Deyrup AT, Jiang W, Luu HH, Luo J, Szatkowski JP, Vanichakarn P, Park JY, Li Y, et al. Characterization of the distinct orthotopic bone-forming activity of 14 BMPs using recombinant adenovirus-mediated gene delivery. Gene Ther. 2004; 11:1312–20. 10.1038/sj.gt.3302298 15269709

[25] Bijsterbosch J, Kloppenburg M, Reijnierse M, Rosendaal FR, Huizinga TW, Slagboom PE, Meulenbelt I. Association study of candidate genes for the progression of hand osteoarthritis. Osteoarthritis Cartilage. 2013; 21:565–69. 10.1016/j.joca.2013.01.011 23357225

[26] Liang W, Gao B, Xu G, Weng D, Xie M, Qian Y. Association between single nucleotide polymorphisms of asporin (ASPN) and BMP5 with the risk of knee osteoarthritis in a Chinese Han population. Cell Biochem Biophys. 2014; 70:1603–08. 10.1007/s12013-014-0102-6 25030405

[27] Snelling SJ, Hulley PA, Loughlin J. BMP5 activates multiple signaling pathways and promotes chondrogenic differentiation in the ATDC5 growth plate model. Growth Factors. 2010; 28:268–79. 10.3109/08977191003752296 20402566

[28] Ro TB, Holt RU, Brenne AT, Hjorth-Hansen H, Waage A, Hjertner O, Sundan A, Borset M. Bone morphogenetic protein-5, -6 and -7 inhibit growth and induce apoptosis in human myeloma cells. Oncogene. 2004; 23:3024–32. 10.1038/sj.onc.1207386 14691444

[29] Rigoglou S, Papavassiliou AG. The NF-κB signalling pathway in osteoarthritis. Int J Biochem Cell Biol. 2013; 45:2580–84. 10.1016/j.biocel.2013.08.018 24004831

[30] Zou J, Lei T, Guo P, Yu J, Xu Q, Luo Y, Ke R, Huang D. Mechanisms shaping the role of ERK1/2 in cellular senescence (review). Mol Med Rep. 2019; 19:759–70. 10.3892/mmr.2018.9712 30535440 PMC6323238

[31] Shih HY, Hsu SY, Ouyang P, Lin SJ, Chou TY, Chiang MC, Cheng YC. Bmp5 regulates neural crest cell survival and proliferation via two different signaling pathways. Stem Cells. 2017; 35:1003–14. 10.1002/stem.2533 27790787

[32] Camarero-Espinosa S, Rothen-Rutishauser B, Foster EJ, Weder C. Articular cartilage: from formation to tissue engineering. Biomater Sci. 2016; 4:734–67. 10.1039/c6bm00068a 26923076

[33] Goldring MB. Articular cartilage degradation in osteoarthritis. HSS J. 2012; 8:7–9. 10.1007/s11420-011-9250-z 23372517 PMC3295961

[34] Goldring MB, Goldring SR. Articular cartilage and subchondral bone in the pathogenesis of osteoarthritis. Ann N Y Acad Sci. 2010; 1192:230–37. 10.1111/j.1749-6632.2009.05240.x 20392241

[35] Elshaier AM, Hakimiyan AA, Rappoport L, Rueger DC, Chubinskaya S. Effect of interleukin-1beta on osteogenic protein 1-induced signaling in adult human articular chondrocytes. Arthritis Rheum. 2009; 60:143–54. 10.1002/art.24151 19116903 PMC2626196

[36] Thielen NG, van der Kraan PM, van Caam AP. TGFβ/BMP signaling pathway in cartilage homeostasis. Cells. 2019; 8:969. 10.3390/cells8090969 31450621 PMC6769927

[37] Lv M, Zhou Y, Polson SW, Wan LQ, Wang M, Han L, Wang L, Lu XL. Identification of chondrocyte genes and signaling pathways in response to acute joint inflammation. Sci Rep. 2019; 9:93. 10.1038/s41598-018-36500-2 30643177 PMC6331554

[38] Szychlinska MA, Castrogiovanni P, Nsir H, Di Rosa M, Guglielmino C, Parenti R, Calabrese G, Pricoco E, Salvatorelli L, Magro G, Imbesi R, Mobasheri A, Musumeci G. Engineered cartilage regeneration from adipose tissue derived-mesenchymal stem cells: a morphomolecular study on osteoblast, chondrocyte and apoptosis evaluation. Exp Cell Res. 2017; 357:222–35. 10.1016/j.yexcr.2017.05.018 28529106

[39] Szychlinska MA, Stoddart MJ, D’Amora U, Ambrosio L, Alini M, Musumeci G. Mesenchymal stem cell-based cartilage regeneration approach and cell senescence: can we manipulate cell aging and function? Tissue Eng Part B Rev. 2017; 23:529–39. 10.1089/ten.TEB.2017.0083 28514935

[40] Kamiya N, Mishina Y. New insights on the roles of BMP signaling in bone-a review of recent mouse genetic studies. Biofactors. 2011; 37:75–82. 10.1002/biof.139 21488130 PMC3551451

[41] Virtanen S, Alarmo EL, Sandström S, Ampuja M, Kallioniemi A. Bone morphogenetic protein -4 and -5 in pancreatic cancer—novel bidirectional players. Exp Cell Res. 2011; 317:2136–46. 10.1016/j.yexcr.2011.06.001 21704030

[42] van Deursen JM. The role of senescent cells in ageing. Nature. 2014; 509:439–46. 10.1038/nature13193 24848057 PMC4214092

[43] Hwang HS, Kim HA. Chondrocyte apoptosis in the pathogenesis of osteoarthritis. Int J Mol Sci. 2015; 16:26035–54. 10.3390/ijms161125943 26528972 PMC4661802

[44] Vakifahmetoglu H, Olsson M, Orrenius S, Zhivotovsky B. Functional connection between p53 and caspase-2 is essential for apoptosis induced by DNA damage. Oncogene. 2006; 25:5683–92. 10.1038/sj.onc.1209569 16652156

[45] Childs BG, Baker DJ, Kirkland JL, Campisi J, van Deursen JM. Senescence and apoptosis: dueling or complementary cell fates? EMBO Rep. 2014; 15:1139–53. 10.15252/embr.201439245 25312810 PMC4253488

[46] Debacq-Chainiaux F, Borlon C, Pascal T, Royer V, Eliaers F, Ninane N, Carrard G, Friguet B, de Longueville F, Boffe S, Remacle J, Toussaint O. Repeated exposure of human skin fibroblasts to UVB at subcytotoxic level triggers premature senescence through the TGF-beta1 signaling pathway. J Cell Sci. 2005; 118:743–58. 10.1242/jcs.01651 15671065

[47] Rebbaa A, Zheng X, Chou PM, Mirkin BL. Caspase inhibition switches doxorubicin-induced apoptosis to senescence. Oncogene. 2003; 22:2805–11. 10.1038/sj.onc.1206366 12743603

[48] Ding L, Heying E, Nicholson N, Stroud NJ, Homandberg GA, Buckwalter JA, Guo D, Martin JA. Mechanical impact induces cartilage degradation via mitogen activated protein kinases. Osteoarthritis Cartilage. 2010; 18:1509–17. 10.1016/j.joca.2010.08.014 20813194 PMC3013628

[49] Jonason JH, Hoak D, O’Keefe RJ. Primary murine growth plate and articular chondrocyte isolation and cell culture. Methods Mol Biol. 2015; 1226:11–18. 10.1007/978-1-4939-1619-1_2 25331039

[50] Culley KL, Dragomir CL, Chang J, Wondimu EB, Coico J, Plumb DA, Otero M, Goldring MB. Mouse models of osteoarthritis: surgical model of posttraumatic osteoarthritis induced by destabilization of the medial meniscus. Methods Mol Biol. 2015; 1226:143–73. 10.1007/978-1-4939-1619-1_12 25331049

[51] Glasson SS, Chambers MG, Van Den Berg WB, Little CB. The OARSI histopathology initiative - recommendations for histological assessments of osteoarthritis in the mouse. Osteoarthritis Cartilage. 2010 (Suppl 3); 18:S17–23. 10.1016/j.joca.2010.05.025 20864019

