# Supplementary material for: BMP5 silencing inhibits chondrocyte senescence and apoptosis as well as osteoarthritis progression in mice
Source: Aging (Albany NY). 2021 Mar 19;13(7):9646–64. doi: 10.18632/aging.202708 (PMC8064147; doi:10.18632/aging.202708)
Supplement: Supplementary Figures [file aging-13-202708-s001.pdf]

SUPPLEMENTARY FIGURES

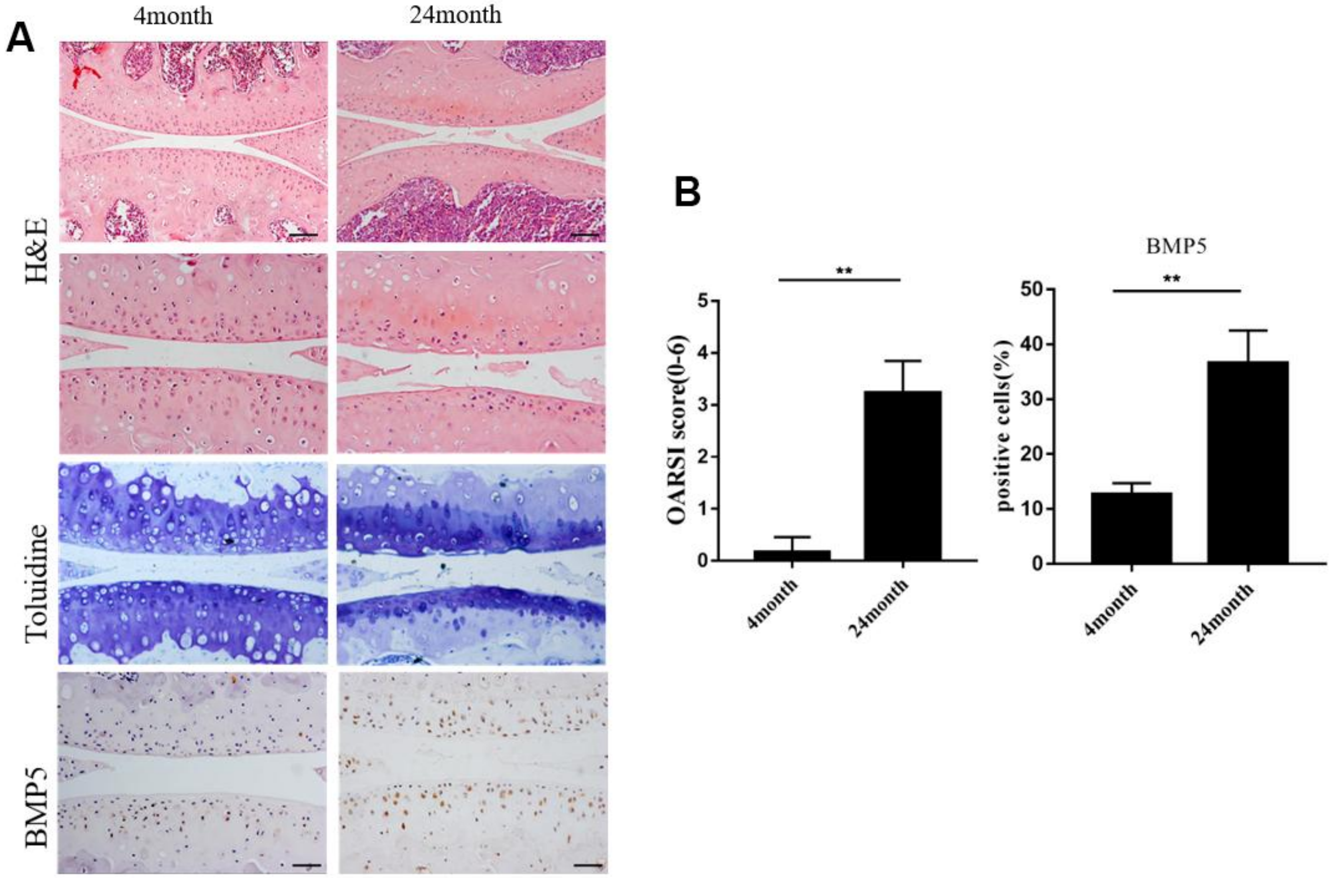

Supplementary Figure 1. BMP5 expression is elevated in chondrocytes from the aged mice. (**A**) Representative images show Safranin O-Fast Green staining and BMP5 immunohistochemical staining in the knee cartilage tissues from young and aged mouse OAA model. Scale bars: 10 μm (first line) and 5 μm (others). (**B**) Quantitative analysis of BMP5 expression and OARSI scores in the DMM mouse model and aged mice are shown. All data are represented as the means ± SD (n = 5 per group). scale bars: 5 μm; **P<0.01.

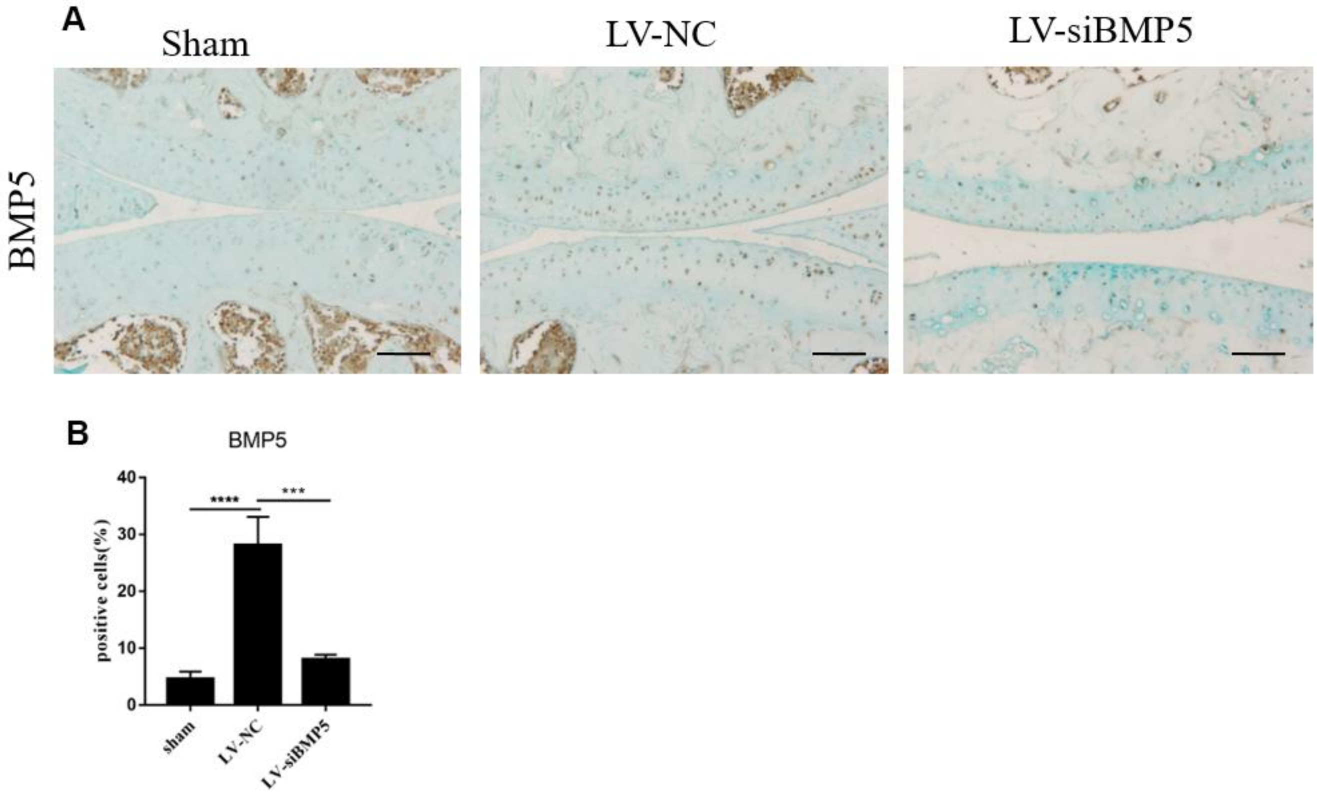

Supplementary Figure 2. Quantitative analysis of lentivirus-mediated BMP5 silencing in DMM model mice. (**A, B**) Representative immunohistochemical (IHC) images and quantitative analysis shows BMP5 expression in the knee articular cartilage tissue sections from sham-operated, DMM plus LV-siNC, and DMM plus LV-siBMP5 groups of mice at 4 weeks post-DMM operation. All data are represented as the means ± SD (n=5 per group); scale bar: 5 μm; ***P<0.01; ****P<0.0001.

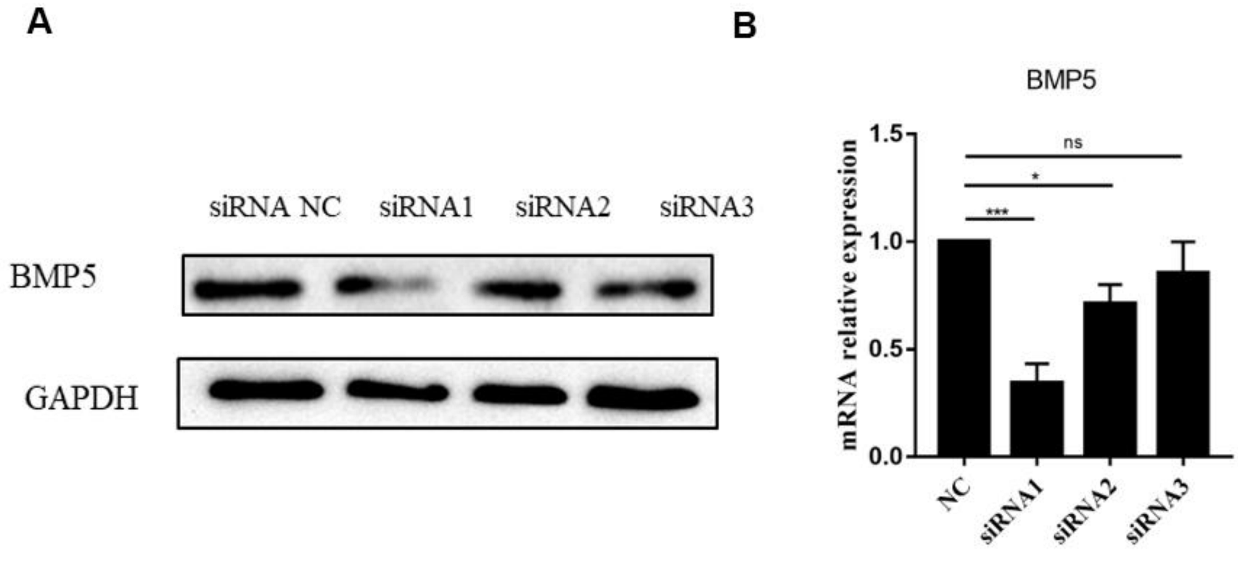

Supplementary Figure 3. Analysis of BMP5 knockdown efficiency of BMP5-targeting siRNAs. (**A**) QRT-PCR (left) and (**B**) western blotting (right) analyses show BMP5 mRNA and protein levels in murine chondrocytes transfected with siNC, BMP5-siRNA1, BMP5-siRNA2, and BMP5-siRNA3, respectively. As shown, the highest knockdown of BMP5 was achieved with BMP5-siRNA1 compared to the siNC control. Note: ns, no significance;*P<0.05; ***P<0.001.

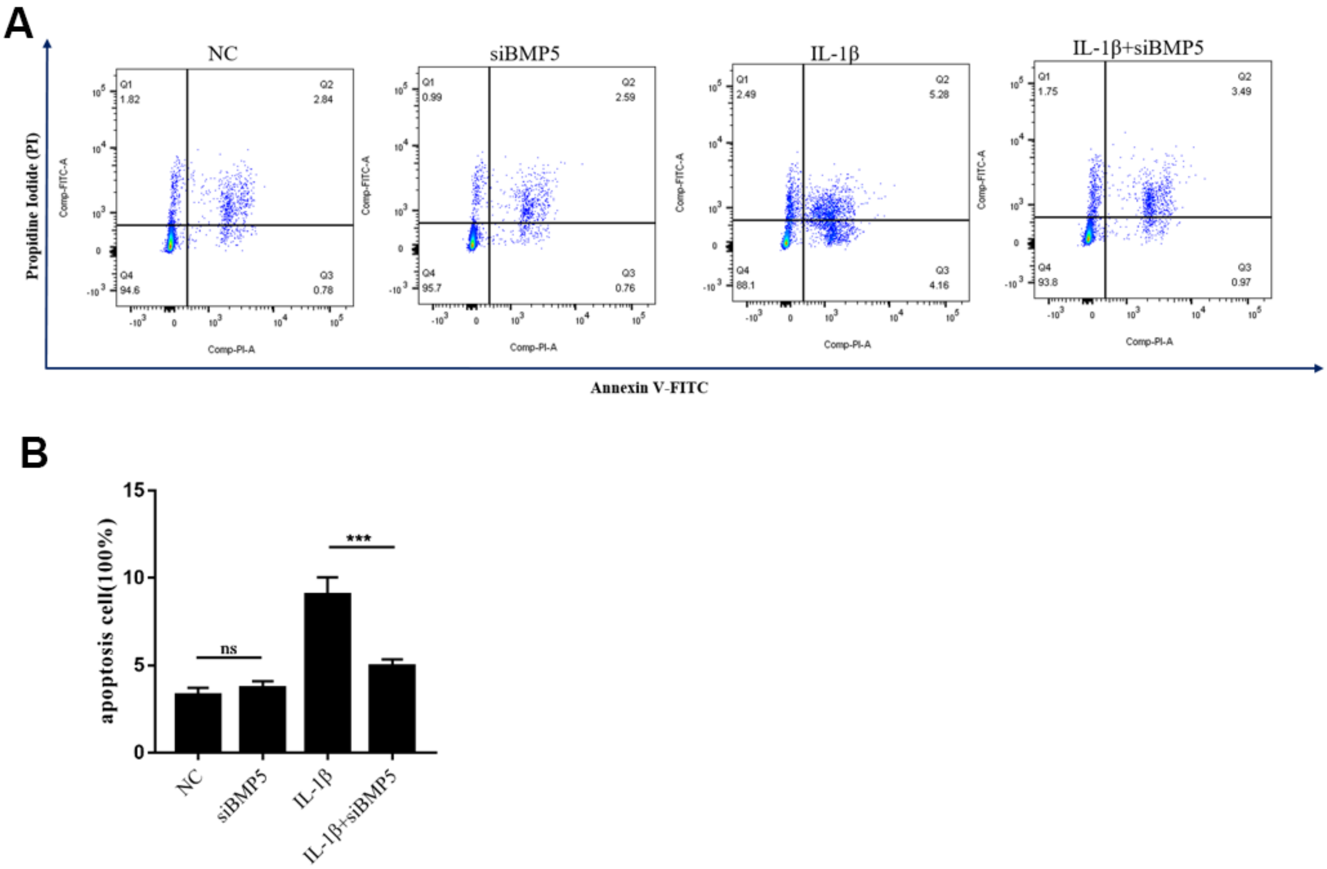

Supplementary Figure 4. BMP5 silencing inhibits IL-1β-induced chondrocyte apoptosis. (**A**, **B**) Flow cytometry assay results show the percentage of apoptotic cells in the siNC- and siBMP5-transfected murine chondrocytes treated with IL-1β and their corresponding controls. Note: ns, no significance; ***P<0.001.
